# Incidence and associated factors of sudden unexpected death in advanced cancer patients: A multicenter prospective cohort study

**DOI:** 10.1002/cam4.4030

**Published:** 2021-06-10

**Authors:** Satoko Ito, Tatsuya Morita, Yu Uneno, Tomohiko Taniyama, Yosuke Matsuda, Hiroyuki Kohara, Isseki Maeda, Takeo Nakayama, Masanori Mori, the EASED Investigators

**Affiliations:** ^1^ Department of Health Informatics Graduate School of Medicine Kyoto University Kyoto Japan; ^2^ Hospice The Japan Baptist Hospital Kyoto Japan; ^3^ Palliative and Supportive Care Division Seirei Mikatahara General Hospital Hamamatsu Japan; ^4^ Department of Therapeutic Oncology Graduate School of Medicine Kyoto University Kyoto Japan; ^5^ Department of Oncology and Palliative Medicine Mitsubishi Kyoto Hospital Kyoto Japan; ^6^ Palliative Care Department St. Luke's International Hospital Tokyo Japan; ^7^ Hiroshima Prefectural Hospital Hiroshima Japan; ^8^ Department of Palliative Care Senri‐Chuo Hospital Toyonaka Japan

**Keywords:** end‐of‐life care, neoplasms, palliative care, prognosis, sudden death

## Abstract

**Purpose:**

A sudden unexpected death has significant negative impacts on patients, family caregivers, and medical staff in hospice/palliative care. This study aimed to clarify the incidence and associated factors of sudden unexpected death according to four definitions in advanced cancer patients.

**Methods:**

We performed a prospective cohort study in 23 inpatient hospices/palliative care units in Japan. Advanced cancer patients aged ≥18 years who were admitted to inpatient hospices/palliative care units were included. The incidence and associated factors of sudden unexpected death were evaluated in all enrolled patients according to four definitions: (a) rapid decline death, defined as a sudden death preceded by functional decline over 1–2 days; (b) surprise death, defined if the primary responsible palliative care physician answered “yes” to the question, “Were you surprised by the timing of the death?”; (c) unexpected death, defined as a death that occurred earlier than the physicians had anticipated; and (d) performance status (PS)‐defined sudden death, defined as a death that occurred within 1 week of functional status assessment with an Australia‐modified Karnofsky PS ≥50.

**Results:**

Among 1896 patients, the incidence of rapid decline death was the highest (30‐day cumulative incidence: 16.8%, 95% CI: 14.8–19.0%), followed by surprise death (9.6%, 8.1–11.4%), unexpected death (9.0%, 7.5–10.8%), and PS‐defined sudden death (6.4%, 5.2–8.0%). Male sex, liver metastasis, dyspnea, malignant skin lesion, and fluid retention were significantly associated with the occurrence of sudden unexpected death.

**Conclusion:**

Sudden unexpected death is not uncommon even in inpatient hospices/palliative care units, with range of 6.4–16.8% according to the different definitions.

## Graphical abstract

A sudden unexpected death has significant negative impacts on patients, family caregivers, and medical staff in palliative care settings. In our prospective multicenter cohort study in inpatient hospices/palliative care units in Japan, we found that the 30‐day cumulative incidence of sudden unexpected death ranged 6.4–16.8% depending on four previously proposed definitions, which revealed that sudden unexpected death is not uncommon.

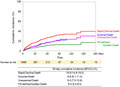

## 1 INTRODUCTION

Advanced cancer patients generally show predictable functional decline in the terminal phase.^1^ Some patients, however, experience an acute event with rapid functional decline, regarded as a sudden unexpected death.^2^ Sudden unexpected death is undesirable for patients who wish to spend their remaining time with their family, or those who want to realize specific wishes.^3^ It also can adversely affect family caregivers’ emotions, and sometimes lead to complicated grief reactions.^4^, ^5^ It also makes decision‐making difficult for healthcare providers.^6^ Understanding sudden unexpected death through clarification of its epidemiology, etiology, and associated factors may help patients and families to prepare for death.

To date, four definitions have been proposed to define sudden unexpected death in palliative care settings with a wide range of incidences from 0.5% to 42%.^7^, ^8^, ^9^, ^10^, ^11^ In small‐scale single‐center retrospective studies, sudden deaths within a few or several days following a rapid decline in functional status occurred at 16–42%,^7^, ^8^, ^9^ while unexpected deaths reported by multidisciplinary team meetings occurred at 0.5–5%.^8^, ^9^ Unexpected deaths were 10% of all deaths in acute palliative care units in a prospective study using surprise question criteria.^10^ Recently, a large‐scale prospective study reported a 4% prevalence of sudden death,^11^ using a definition based on Australia‐modified Karnofsky Performance Status (AKPS) scores.^12^ We reasoned that these discrepancies in the frequency of sudden unexpected death are due to the use of different definitions among studies in addition to differences in patients’ characteristics and settings. The absence of a unified definition of sudden unexpected death makes it difficult to understand its frequency and predictors.

The primary aim of this study was to clarify the incidence of sudden unexpected death in patients with advanced cancer using the four proposed definitions in the same cohort. We also explored their agreement in the identified cases and additional associated factors.

## 2 PATIENTS AND METHODS

### 2.1 Study design and patients

This study was conducted as a part of a multicenter prospective cohort study in a convenience sample of 23 inpatient hospices/palliative care units in Japan between January 2017 and June 2018. The participating institutions comprised certified inpatient hospices/palliative care units recruited from among 394 institutions throughout the country. Consecutive eligible patients were enrolled if they had been admitted to the participating inpatient hospices/palliative care units between January and December 2017. The inclusion criteria of this study were: (a) adult patients (aged ≥18 years), (b) patients diagnosed with locally extensive or metastatic cancer, and (c) patients admitted to inpatient hospices/palliative care units. If patients were hospitalized more than once, only data from their first hospitalization during the study period were collected, regardless of the number of hospitalizations. Patients who were scheduled to be discharged within a week or did not want to participate were excluded. Patients were consecutively enrolled; however, with limited medical resources, in order to reduce the burden on researchers, we allowed each institution to select the days or terms of registration in advance. Therefore, we registered all patients who were admitted on these days or during the defined periods unselectively and consecutively, regardless of patient characteristics or general condition. All enrolled patients were followed up to 6 months or until death. Patients discharged alive were not followed, because they were usually treated by physicians of nonparticipating institutions.

### 2.2 Data collection

Primary responsible palliative care physicians collected baseline data including age, sex, primary and metastatic cancer sites, comorbidities,^13^ medications, and performance status (PS) on admission. PS was measured using the Eastern Cooperative Oncology Group Performance Status (ECOG‐PS), Karnofsky Performance Status (KPS),^14^ Palliative Performance Status (PPS),^15^ and palliative care phase.^16^ Symptoms including pain, dyspnea, fatigue, and anorexia were assessed according to the Japanese version of the Integrated Palliative Care Outcome Scale (IPOS).^17^, ^18^ In addition, fluid retention symptoms (bilateral extremity edema, pleural effusion, or ascites); malignant gastrointestinal obstruction; malignant skin lesion defined as skin cancer, direct invasion to skin, or metastatic skin lesion; and delirium were diagnosed based on the Diagnostic and Statistical Manual of Mental Disorders, 5th edition. In general, primary responsible physicians see patients at least twice a day and confirm patients’ death in inpatient hospices/palliative care units in Japan. Palliative care physicians involved in this study were familiar with scoring on those measures in routine practice, and they completed a brief education session for data collection before initiation of the study.

For patients who died in hospices/palliative care units, primary responsible palliative care physicians assessed whether their death was a sudden unexpected death using four definitions: (a) rapid decline death, defined as a sudden death preceded by rapid (1–2 days) functional decline ^8^ ; (b) surprise death, defined if the primary responsible palliative care physician answered “yes” to the question, “Were you surprised by the timing of the death?” (surprise question) ^10^ ; (c) unexpected death, defined as a death that occurred earlier than expected by the primary responsible palliative care physician ^8^, ^9^ ; and (d) PS‐defined sudden death, defined as a sudden death that occurred within 1 week of functional status assessment with an AKPS of 50 or greater.^11^, ^19^ Immediately after the patients’ death, the primary responsible physicians evaluated the AKPS 1 week before the patients’ death. The AKPS was recorded on the basis of medical records if they had been outpatients or in general wards in the same hospital. When information regarding the period before admission to hospices/palliative care units was unavailable, we used the KPS on admission because the KPS is highly consistent with AKPS.^12^

Primary responsible physicians reported definite and probable complications that directly contributed to death in all cases. Definite complications included obvious causes, such as bleeding and suffocation, confirmed by confirmatory examinations or autopsy; and probable complications were those assessed by non‐confirmatory examinations.

### 2.3 Statistical analysis

Baseline demographic data were summarized using descriptive statistics. Cumulative incidences of sudden unexpected death according to each definition were analyzed using the Kaplan–Meier method. Data from patients who were discharged alive from inpatient hospices/palliative care units and those who did not experience sudden unexpected death were censored. Agreement among definitions was assessed using kappa statistics. We also calculated the frequency of sudden unexpected death among patients who died within 6 months in inpatient hospices/palliative care units for each definition. To explore the factors associated with sudden unexpected death, we calculated hazard ratios using a Cox regression model. The model included 18 variables: sex, age, primary cancer sites (gastrointestinal tract, hepatobiliary tract/pancreas, lung, or other), metastatic sites (liver, lung, bone, or brain), ECOG‐PS on admission, IPOS ≥2 symptoms on admission (pain, dyspnea, fatigue, or anorexia), clinical findings (malignant skin lesion, gastrointestinal obstruction, fluid retention, or delirium), and comorbidities (cardiovascular disease or chronic lung disease). We calculated 95% confidence intervals (CI) for all estimates. A *p*‐value <0.05 was considered significant. We elected not to make adjustments for multiple comparisons, due to the completely exploratory nature of this study. Statistical analyses were performed using JMP® Pro 13 (SAS Institute Inc.).

## 3 RESULTS

### 3.1 Patient characteristics

**FIGURE 1:**
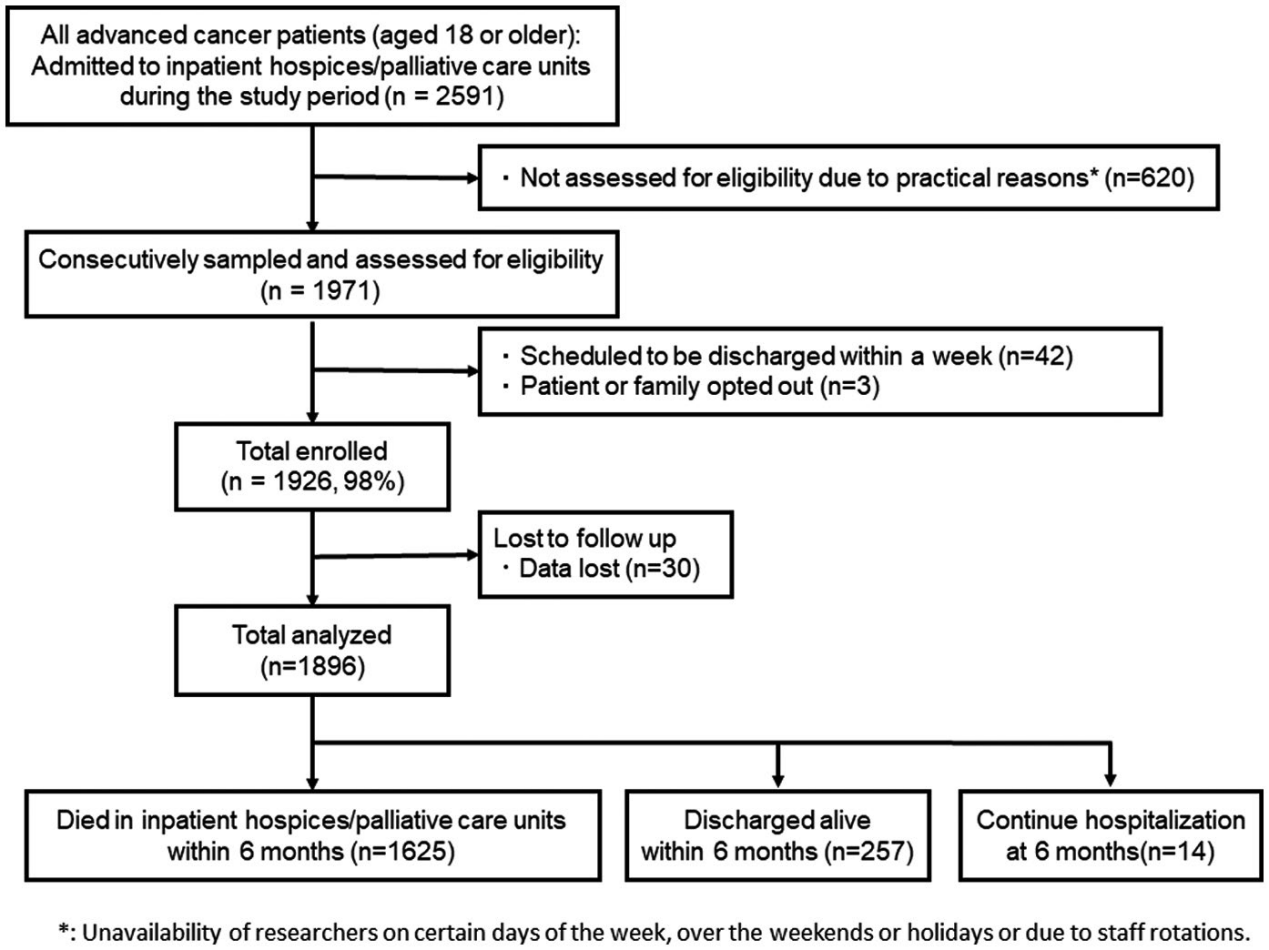
Flow chart of participant enrollment

Figure 1 shows the flowchart of this cohort study. Of the 2591 patients admitted to inpatient hospices/palliative care units during our study period, a total of 1971 patients were assessed for eligibility consecutively. Of these, 1926 patients were included (98% enrollment rate). As data from 30 of these patients were missing, we analyzed a total of 1896 patients: 1625 died in the hospice/palliative care unit, 257 were discharged alive, and 14 continued to be hospitalized.

Patient characteristics are summarized in Table 1. Mean age was 72.4 years, and 965 patients (51%) were male. The most common primary cancer site was the gastrointestinal tract (28%), followed by the hepatobiliary tract and pancreas (19%). Most patients had ECOG‐PS 3 or 4, with median KPS and PPS scores of 40 and 40, respectively. Median length of stay was 17 days.

**TABLE 1:** Patient characteristics at study enrollment

Characteristics	n = 1896	
Age, mean ± SD (years old)	72.4 ± 12.3	
Male	965	(51%)
Primary tumor site		
Gastrointestinal tract	527	(28%)
Hepatobiliary tracts and pancreas	363	(19%)
Lung	319	(17%)
Genitourinary system	141	(7%)
Breast	131	(7%)
Gynecologic organs	119	(6%)
Head and neck	106	(6%)
Blood and lymph nodes	56	(3%)
Others	134	(7%)
Metastatic site		
Liver	730	(39%)
Lung	708	(37%)
Bone	501	(26%)
Brain	263	(14%)
Comorbidity		
Cardiovascular disease	110	(6%)
Chronic lung disease	118	(6%)
ECOG PS		
0 / 1 / 2 / 3 / 4	3/23/158/797/915	(0.2 / 1 / 8 / 42 / 48%)
KPS, median (range)	40 (10–90)	
≥50, 30–40, 10–20	572 / 978 / 345	(30 / 52 / 18%)
PPS, median (range)	40 (10–90)	
≥60, 30–50, 10–20	184 / 1363 / 349	(10 / 72 / 18%)
Palliative care phase		
Stable/ Unstable/ Deteriorating/ Terminal	177 / 579 / 921 / 217	(9 / 31 / 49 / 11%)
Symptoms		
Pain (IPOS ≥2)	664	(35%)
Dyspnea (IPOS ≥2)	380	(20%)
Fatigue (IPOS ≥2)	787	(42%)
Anorexia (IPOS ≥2)	865	(49%)
Gastrointestinal obstruction	257	(14%)
Malignant skin lesion^a^	156	(8%)
Delirium	583	(31%)
Fluid retention	1219	(64%)
Observation period in days, average, median (range)	27, 17 (1–180)	

Abbreviations: ECOG PS, Eastern Cooperative Oncology Group Performance status; IPOS, Integrated Palliative care Outcome Scale; KPS, Karnofsky Performance Status; PPS, Palliative Performance Status; SD, standard deviation.

^a^ Malignant skin lesion: primary skin cancer (n = 11), metastatic skin lesion or direct invasion to skin (n = 145).

### 3.2 Cumulative incidences of sudden unexpected death according to the four definitions

**FIGURE 2:**
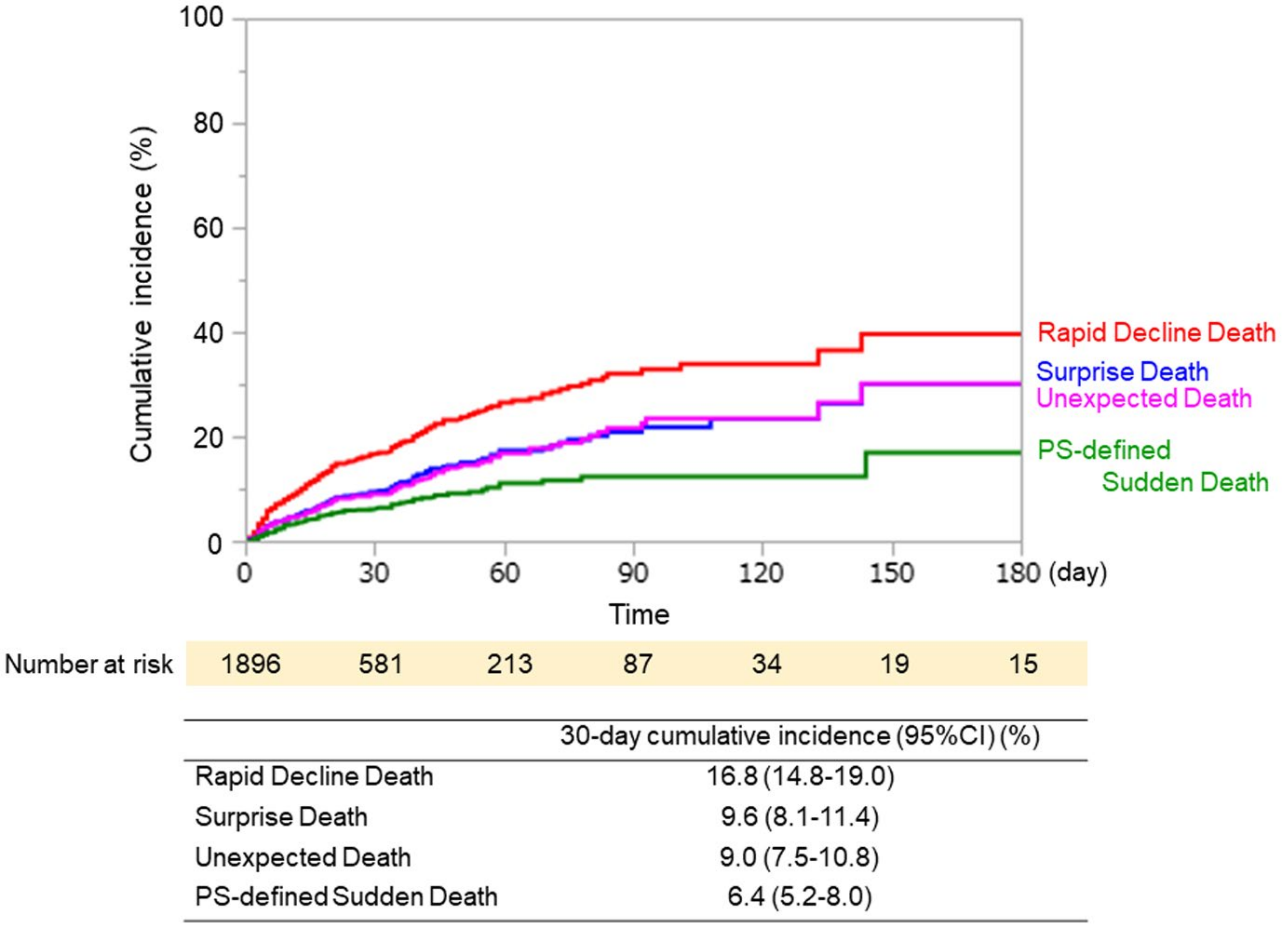
(Top) Cumulative incidences of sudden unexpected death were estimated by the Kaplan‐Meier method. Patients who were discharged alive from inpatient hospices/palliative care units and those who did not experience sudden unexpected death were censored. (Bottom) The 30‐day cumulative incidences and 95% CI (confidence intervals) according to four definitions are shown

Figure 2 shows the cumulative incidences of sudden unexpected death according to the four definitions. The cumulative incidence of rapid decline death was the highest (30‐day cumulative incidence [95% CI]: 16.8% [14.8–19.0%]), followed by surprise death (9.6% [8.1–11.4%]), unexpected death (9.0% [7.5–10.8%]), and PS‐defined sudden death (6.4% [5.2–8.0%]). AKPS scores 1 week before death could not be evaluated and were replaced with KPS at the time of admission in 29 patients when assessing PS‐defined sudden death.

### 3.3 Agreement among the four definitions

Table 2 summarizes agreement among the four definitions. There was high agreement between surprise death and unexpected death (kappa [95% CI]: 0.77 [0.72–0.82]), and moderate agreement between rapid decline death and surprise death (0.56 [0.50–0.62]) or unexpected death (0.53 [0.47–0.59]). On the other hand, there was low agreement between PS‐defined sudden death and rapid decline death (0.19 [0.13–0.25]), surprise death (0.18 [0.11–0.25]), or unexpected death (0.19 [0.12–0.26]).

**TABLE 2:** Agreement among four definitions (kappa statistics)

	κ(95%CI)		
	Surprise death	Unexpected death	PS‐defined sudden death
Rapid decline death	0.56 (0.50–0.62)	0.53 (0.47–0.59)	0.19 (0.13–0.25)
Surprise death	—	0.77 (0.72–0.82)	0.18 (0.11–0.25)
Unexpected death	—	—	0.19 (0.12–0.26)
PS‐defined sudden death	—	—	—

Abbreviation: CI, confidence interval

— indicates overlapping data

### 3.4 Complications clinically assumed to contribute to death

Table 3 presents complications clinically assumed to contribute to death. Commonly observed complications included aspiration/suffocation, pulmonary embolism, gastrointestinal bleeding, and infection. Most complications were assumed based on non‐confirmatory examinations by primary responsible physicians, and complications were definitely diagnosed in 19 patients.

**TABLE 3:** Frequencies of sudden unexpected death and complications clinically assumed to contribute to death^a^

	All deaths		Rapid decline death		Surprise death		Unexpected death		PS‐defined sudden death	
Frequency	n = 1625		n = 295/1625 (18%)		n = 168/1625 (10%)		n = 164/1625 (10%)		n = 104/1625 (6%)	
Complications leading to death	n	(%)	n	(%)	n	(%)	n	(%)	n	(%)
Aspiration / Suffocation	28	(2)	24	(8)	16	(10)	14	(8)	1	(1)
Pulmonary embolism	13	(1)	13	(4)	12	(7)	10	(6)	3	(3)
Gastrointestinal bleeding	19	(1)	9	(3)	2	(1)	3	(2)	2	(2)
Infection	19	(1)	8	(3)	6	(4)	7	(4)	3	(2)
Gastrointestinal perforation	9	(1)	4	(1)	4	(2)	4	(2)	4	(4)
Cerebrovascular disease	8	(1)	4	(1)	4	(2)	3	(2)	0	(0)
Intraabdominal hemorrhage	4	(1)	4	(1)	3	(2)	3	(2)	1	(1)
Pulmonary hemorrhage	4	(1)	4	(1)	0	(0)	0	(0)	0	(0)
Myocardial infarction	3	(1)	2	(1)	1	(1)	1	(1)	1	(1)
Others	11^b^	(1)	6^c^	(2)	6^c^	(4)	6^c^	(4)	1^c^	(1)
Total	118	(7)	78	(26)	54	(32)	51	(31)	16	(15)

^a^ The complications were assumed by the primary responsible palliative care physicians to contribute directly to death.

^b^ External bleeding due to malignant skin lesion, senility (n = 2, each), genital bleeding, interstitial pneumonia, pneumothorax, heart failure, ileus, diarrhea, abnormal electrolyte (n = 1, each).

^c^ External bleeding due to malignant skin lesion, genital bleeding, interstitial pneumonia, pneumothorax, heart failure, ileus, diarrhea (n = 1, each).

### 3.5 Associated factors of sudden unexpected death

Table 4 shows associated factors of sudden unexpected death identified by multivariate Cox regression analysis. For rapid decline death and surprise death, the following five independent factors were identified: dyspnea, malignant skin lesion, liver metastasis, male sex, and fluid retention. For unexpected death, malignant skin lesion, fluid retention, gastrointestinal obstruction, male sex, and liver metastasis were identified as associated factors. For PS‐defined sudden death, dyspnea, good PS, and absence of brain metastases were identified as associated factors. Collectively, male sex, dyspnea, liver metastasis, malignant skin lesion, and fluid retention were consistently associated with at least three definitions.

**TABLE 4:** Associated factors of sudden unexpected death in palliative care identified by multivariate analyses

	Rapid decline death				Surprise death				Unexpected death				PS‐defined sudden death			
	HR	95%CI		*p*	HR	95%CI		*p*	HR	95%CI		*p*	HR	95%CI		*p*
Male	**1.44**	**1.12**	**1.85**	**0.004**	**1.61**	**1.16**	**2.24**	**0.005**	**1.60**	**1.15**	**2.23**	**0.005**	1.22	0.80	1.87	0.35
<70 years old	1.00	0.77	1.29	0.99	0.94	0.67	1.31	0.73	0.89	0.63	1.24	0.49	1.34	0.88	2.04	0.17
Primary cancer site																
Gastrointestinal tracts	0.92	0.67	1.27	0.61	0.93	0.61	1.40	0.72	1.08	0.71	1.63	0.73	0.69	0.40	1.17	0.17
Hepatobiliary tracts/Pancreas	1.17	0.82	1.67	0.39	1.08	0.67	1.74	0.74	1.12	0.69	1.83	0.65	0.85	0.46	1.54	0.61
Lung	1.18	0.80	1.72	0.40	1.07	0.63	1.81	0.80	1.32	0.78	2.25	0.30	1.32	0.71	2.46	0.38
Others	1.00				1.00				1.00				1.00			
ECOG PS 3–4	1.34	0.89	2.01	0.16	1.06	0.66	1.71	0.80	1.09	0.67	1.78	0.72	**0.34**	**0.21**	**0.56**	**<.001**
Comorbid of cardiovascular disease	1.22	0.77	1.86	0.38	1.28	0.69	2.17	0.41	0.90	0.44	1.66	0.76	0.80	0.28	1.81	0.62
Comorbid of chronic lung disease	1.31	0.83	1.98	0.24	1.00	0.49	1.83	1.00	0.85	0.40	1.59	0.62	0.87	0.35	1.84	0.73
Liver metastasis	**1.60**	**1.23**	**2.06**	**<.001**	**1.64**	**1.16**	**2.31**	**0.005**	**1.49**	**1.05**	**2.10**	**0.025**	1.43	0.91	2.23	0.12
Lung metastasis	0.99	0.76	1.28	0.95	1.07	0.76	1.51	0.68	0.99	0.70	1.39	0.94	0.90	0.57	1.40	0.66
Bone metastasis	1.01	0.76	1.35	0.92	0.79	0.53	1.16	0.23	0.78	0.51	1.16	0.22	0.72	0.41	1.20	0.21
Brain metastasis	0.90	0.59	1.33	0.61	1.00	0.57	1.67	0.99	1.03	0.58	1.73	0.92	**0.30**	**0.10**	**0.72**	**0.005**
Pain (IPOS ≥2)	1.05	0.82	1.35	0.68	1.38	0.99	1.91	0.055	1.24	0.89	1.72	0.21	0.86	0.55	1.31	0.48
Dyspnea (IPOS ≥2)	**2.02**	**1.51**	**2.69**	**<.001**	**1.55**	**1.01**	**2.33**	**0.045**	1.53	0.99	2.30	0.055	**1.89**	**1.14**	**3.05**	**0.014**
Fatigue (IPOS ≥2)	1.06	0.81	1.40	0.67	0.98	0.67	1.41	0.90	1.15	0.79	1.65	0.47	1.28	0.80	2.05	0.31
Anorexia (IPOS ≥2)	1.18	0.90	1.55	0.24	1.15	0.80	1.65	0.44	1.04	0.72	1.50	0.82	1.47	0.92	2.36	0.11
Gastrointestinal obstruction	1.36	0.96	1.89	0.080	1.39	0.89	2.12	0.14	**1.68**	**1.10**	**2.52**	**0.017**	0.97	0.51	1.72	0.92
Malignant skin lesion	**1.67**	**1.12**	**2.43**	**0.014**	**1.97**	**1.19**	**3.11**	**0.009**	**1.75**	**1.02**	**2.83**	**0.041**	1.06	0.48	2.05	0.88
Fluid retention	**1.40**	**1.07**	**1.83**	**0.012**	**1.61**	**1.14**	**2.31**	**0.007**	**1.70**	**1.19**	**2.45**	**0.003**	1.22	0.79	1.91	0.38
Delirium	0.88	0.65	1.17	0.38	0.76	0.50	1.13	0.18	0.76	0.50	1.13	0.18	1.19	0.71	1.93	0.50

Abbreviations: CI, confidence interval; ECOG PS, Eastern Cooperative Oncology Group Performance Status; HR, Hazard Ratio; IPOS, Integrated Palliative care Outcome Scale.

## 4 DISCUSSION

In this study, we prospectively investigated the incidence of sudden unexpected death of advanced cancer patients using four proposed definitions as well as associated factors and complications clinically assumed to contribute to death. The novelty of this study was that we compared the incidence using four proposed definitions in a single cohort.

Most importantly, we found that 30‐day cumulative incidences of sudden unexpected death ranged from 6.4 to 16.8% and confirmed the finding that sudden unexpected death is not uncommon. For comparisons, we calculated the frequency of sudden unexpected death using previously reported studies.^7^, ^8^, ^9^, ^10^, ^11^ The frequency of rapid decline death and unexpected death was calculated to be 18% and 10%, respectively, in our cohort, while these figures are close to the 16% and 5%, respectively, reported in a previous retrospective study in an inpatient hospice in the UK.^8^ The frequency of surprise death was 10% both in our study and in a previous study of patients admitted to two acute palliative care units in the US and Brazil, despite the difference in settings.^10^ In addition, the frequency of PS‐defined sudden death was 6% in the current study, comparable to that reported by Ekstrom et al. (4%), who enrolled non‐cancer patients and those in home hospice.^11^ Furthermore, our participating institutions fairly represent all certified inpatient hospices/palliative care units in Japan, because the percentage of patients who died (84% in national data vs. 84% [1625/1926 patients] in this sample) and the average length of stay (32 days in national data vs. 27 days in this sample) are consistent with our results.^20^ The median length of hospice stays in the US (19 days^21^) and in the other countries (4–23 days^22^) are also similar to that in our study (17 days), although our study included a certain number of patients, with better PS and long‐term survival. Thus, our results may generalize not only to patients in hospices/palliative care units in Japan, but also inpatient hospices in other countries, even though careful consideration is necessary.

The second important finding was that the incidence of sudden unexpected death varied by definitions. Rapid decline death had the highest incidence, followed by surprise death, unexpected death, and PS‐defined sudden death. This result is consistent with previous studies showing that the frequency of rapid decline death was relatively high, whereas that of PS‐defined sudden death was low.^7^, ^11^ The high agreement between surprise death and unexpected death are likely due to the similarity in these two definitions. On the other hand, PS‐defined sudden death, the most objective definition, had low agreement with the other three definitions. This is potentially because patients with low AKPS scores at baseline who died from an unexpected acute fatal event were not regarded as sudden death using this definition. PS‐defined sudden death may therefore underestimate the incidence of sudden unexpected death from a subjective point of view. Our results revealed that variations in the incidences of sudden unexpected death depended on their definitions and future studies are needed to characterize which definition is better in evaluating a specific question.

We also explored factors associated with sudden unexpected death. In rapid decline death, surprise death, and unexpected death, we identified new associated factors, including malignant skin lesion, liver metastasis, and fluid retention, in addition to previously reported predictors such as male sex and dyspnea.^11^ Among patients with malignant skin lesions, only two patients with skin invasion in head and neck cancer died due to fatal external bleeding. While this finding suggests that overall tumor burden rather than massive bleeding may affect sudden unexpected death, the clinical implication of malignant skin lesions in association with sudden unexpected death needs further investigation due to the small sample size.

This study has some limitations. First, patients who were discharged alive were analyzed as censored cases. The survival time of these patients was relatively longer (median, 67 days; range, 2–178 days) than that of all enrolled patients (median, 17 days), which indicates that patients discharged alive could be in better condition. Considering that those who are in better condition are possibly at risk of sudden unexpected death, the incidence of sudden unexpected death might be underestimated. Second, we only included and assessed patients who were admitted for the first time during the enrollment period regardless of the number of hospitalizations. The enrolled patients who had been discharged alive and analyzed as censored cases were not included again when they deteriorated and were readmitted to the hospice. The exclusion of these patients could result in a decreased proportion of patients with worse condition who are unlikely to experience sudden unexpected death. Therefore, the incidence of sudden unexpected deaths was potentially overestimated by excluding subsequent admissions. Third, the physician's prognosis was generally inaccurate and optimistic^23^; therefore, future studies may want to include other healthcare providers. Fourth, as there might be a discordance among performance scale measures^24^ and a considerable number of the patients had a high PS, PS‐based classification systems for sudden death might be inaccurate. Fifth, complications contributing to death were mostly clinically diagnosed, and these complications may be under or misdiagnosed. Sixth, we used variables acquired at the time of admission and did not consider time‐dependent factors in evaluating the predictors. Finally, as the patients studied were limited to those admitted to inpatient hospices/palliative care units, the results may not be generalizable to other patient groups.

## 5 CONCLUSIONS

Sudden unexpected deaths are not uncommon even in inpatient hospices/palliative care units, with a range of 6.4–16.8% according to different definitions. Clinically, it might be important for palliative care physicians and clinical oncologists to communicate with patients and families, keeping in mind the possibility of sudden unexpected death. Further studies are needed to characterize, which definition is better for evaluating different situations.

## 6 ETHICAL APPROVAL STATEMENT

This study was conducted in accordance with the ethical standards set forth in the Helsinki Declaration and ethical guidelines for medical and health research involving human subjects issued by the Ministry of Health, Labour and Welfare in Japan. Japanese law does not require individual informed consent from participants in a non‐invasive observational trial; thus, we used an opt‐out method. All institutions were asked to convey information on the study to patients and their families, through a homepage or written materials, such that patients would be able to refuse to participate or withdraw from the study. This study was approved by the Kyoto University Graduate School and Faculty of Medicine Ethics Committee (approval no. R0830) and the local ethics committee of each participating institution.

## CONFLICTS OF INTEREST

T. Nakayama reports personal fees from Otsuka Pharmaceutical Co., other from Nakamura hospital, other from Japan Medical Data Center, personal fees from Dainippon Sumitomo Pharmaceutical Co., personal fees from Ono Pharmaceutical Co., personal fees from Chugai Pharmaceutical Co., personal fees from Dentsu Co., personal fees from Takeda Pharmaceutical Co., personal fees from Novo Nordisk Pharma. Co., personal fees from Janssen Pharmaceutical K.K., personal fees from Boehringer Ingelheim International GmbH, other from Hanshin Dispensing Holding Co., Ltd., personal fees from Pfizer Japan Inc., personal fees from Nikkei Business Publications, Inc., personal fees from Eli Lilly Japan K.K., personal fees from Baxter, and personal fees from Alexion, outside the submitted work. All remaining authors have declared no conflicts of interest.

## AUTHOR CONTRIBUTIONS

Satoko Ito: Conceptualization, formal analysis, investigation, writing‐original draft, and writing‐review and editing. Tatsuya Morita: Conceptualization, investigation, supervision, writing‐original draft, and writing‐review and editing. Yu Uneno, Tomohiko Taniyama, Yosuke Matsuda, Hiroyuki Kohara, and Isseki Maeda: Investigation, and writing‐review and editing. Takeo Nakayama: Conceptualization, formal analysis, supervision, writing‐original draft, and writing‐review and editing. Masanori Mori: Conceptualization, data curation, funding acquisition, investigation, project administration, supervision, and writing‐review and editing.

## Data Availability

Individual deidentified participant data that underlie the results reported in this article will be shared with researchers who provide a methodologically sound proposal. Proposals should be directed to the corresponding author.
